# Different mutational characteristics of the subsets of EGFR-tyrosine kinase inhibitor sensitizing mutation-positive lung adenocarcinoma

**DOI:** 10.1186/s12885-018-5116-9

**Published:** 2018-12-06

**Authors:** Eun Young Kim, Arum Kim, Gaeun Lee, Hangsuck Lee, Yoon Soo Chang

**Affiliations:** 1 Department of Internal Medicine, Yonsei University College of Medicine, 50-1 Yonsei-ro, Seodaemun-gu, Seoul, Republic of Korea; 2 Department of Mathematics, Sungkyunkwan University, 25-2 Sungkyunkwan-ro, Jongno-gu, Seoul, Republic of Korea; 3 Department of Internal Medicine, Yonsei Univeristy College of Medicine, 4th Floor Research Center for Future Medicine, 63-Gil 20, Eonju-ro, Gangnam-gu, Seoul, Republic of Korea

**Keywords:** EGFR, Lung adenocarcinoma, Mutational signatures, Next-generation sequencing, Sensitizing mutation, Tyrosine kinase inhibitor

## Abstract

**Background:**

A subset of lung adenocarcinoma with EGFR-tyrosine kinase inhibitor sensitizing mutations (mEGFR) is common in non-smokers and women, suggesting that mutational stressors other than smoking are involved.

**Methods:**

Targeted sequencing using a custom panel containing 70 cancer-related genes were performed from 73 cases of lung adenocarcinoma with mEGFR (study cohort). In parallel, publicly available data of 47 TCGA-LUAD cases with mEGFR (LUAD cohort) were extracted from the GDC data portal and analyzed by non-negative matrix factorization using the Maftools package.

**Results:**

In the study cohort, the C > A transversions accounted for 12.9% of all single nucleotide variations (SNVs), comprising the second smallest proportion among SNVs. The E19del-subgroup had a significantly lower mutational burden with significantly higher Ti/Tv ratio than the SNV-subgroup, which includes cases with L858R and other EGFR-TKI sensitizing SNVs. (*P* = 0.0326 and 0.0002, respectively, Mann-Whitney U test). In the LUAD cohort, the mutational burden was substantially lower than in other TCGA cancer cohorts, and the frequency of C > A transversions was 30.3%, occupying the second frequency. The E19del-subgroup had a lower mutational burden overall and a higher Ti/Tv ratio than the SNV-subgroup (*P* = 0.0497 and *P* = 0.0055, respectively, Mann-Whitney U test). Smoking-related signature 4 was observed only in the L858R-subgroup, while ignature 30 and 5 was observed in both groups.

**Conclusions:**

Lung adenocarcinoma with mEGFR(+) has a lower mutational burden and does not show a characteristic mutation pattern influenced by smoking. E19del and L858R, which are representative subtypes of mEGFR(+) lung adenocarcinoma, differ in terms of mutational spectrum, as the E19del-subgroup has a lower mutation burden and a higher Ti/Tv ratio than the SNV-subgroup. These findings could help explain the differences in the responses to EGFR-TKIs and in the clinical courses between the two lung adenocarcinoma subgroups.

**Electronic supplementary material:**

The online version of this article (10.1186/s12885-018-5116-9) contains supplementary material, which is available to authorized users.

## Background

According to the 2015 annual report, 1,824,700 new lung cancer cases are diagnosed each year, which accounts for 13% of all cancers, excluding non-melanoma skin cancers. In addition, it is still the leading cause of cancer mortality worldwide, suggesting that lung cancer is a major problem for healthcare worldwide [1]. According to the 2015 yearbook of the National Cancer Registration and Statistics in Korea, lung cancer occurred in 66.0 per 100,000 males and 28.7 per 100,000 females [2]. Compared to other countries, Korea is 10th in the incidence of male lung cancer and 4th in the incidence of female lung cancer [3]. The total incidences in Korea are not significantly different from other countries, but the incidence of lung cancer is higher in non-smokers and women in Korea, and e*pidermal growth factor receptor* (EGFR) mutations are detected much more frequently than in Western countries.

The main causes of lung cancer are direct and indirect smoking, radon, indoor emissions from household combustion, and exhaust from diesel engines (https://monographs.iarc.fr/agents-classified-by-the-iarc/) [4]. Mutational analysis of lung cancer using publicly available data such as TCGA has shown that the smoke-related signature, with many C > A transversions, is a dominant signature in lung adenocarcinoma and lung squamous cell carcinoma [5]. Somatic mutations in cancer are caused infidelity of the DNA replication machinery as well as and defects in DNA repair mechanisms following exposure to endogenous or exogenous mutagens [6]. The somatic mutations observed in some cancers are significantly related to exposure to a specific carcinogen, such as smoking in lung cancer and ultraviolet light in skin cancer [7].

Certain mutational processes in cancer often accompany unique combinations of mutation types called signatures [8, 9]. Recently, Alexandrov et al. developed a theoretical model and computational framework that could deconstruct unique patterns of somatic mutations using cancer specimen sequencing data based on the analysis of somatic substitutions obtained from whole genome sequencing of breast cancer patients [10, 11]. Among the 30 signatures, signature 4, which is characterized by a majority of C > A mutations along with some other base substitution classes, is found only in cancer types in which smoking is a major risk factor and in epithelial cancers that are directly exposed to cigarette smoke. The mutational signature is similar to the mutational pattern resulting from exposing cells to benzo [a] pyrene, a major carcinogen in tobacco. This mutational pattern occurs in the process of nucleotide excisional repair after binding of a bulky DNA adduct to the guanine [5, 8].

Given that lung adenocarcinoma with EGFR-tyrosine kinase inhibitor (TKI) sensitizing mutation (mEGFR) is common in light and/or non-smokers, Asians, and women, it is expected to have mutational pressures other than cigarette smoking, [12]. The L858R mutation and the exon 19 deletion (E19del), which includes the LREA motif, comprise up to 90% of EGFR mutations, followed by L861Q, G719X, and rare mutations [13]. The clinical outcomes of mEGFR positive lung cancers have dramatically improved due to the development of target drugs, but these cancers eventually acquire drug resistance and show disease progression [14, 15]. The clinical courses and responses to EGFR-TKIs differ between the E19del and L858R groups, which are representative subtypes of mEGFR [16]. Therefore, the identification of carcinogenesis by the estimation of significant mutagenic stressors is needed, along with a proactive approach for these subtypes of cancer.

Therefore, we investigated whether there is a distinctive mutation pattern in the subtypes of lung adenocarcinoma responsive to EGFR-TKI. Targeted sequencing was performed on major cancer-related genes using lung adenocarcinoma with mEGFR (study cohort), and the characteristics of the obtained mutations were analyzed. In addition, the mutation characteristics of the L858R and E19del subtypes, which occupy the majority of the mEGFR, were compared and analyzed. Finally, whole exome sequencing data from TCGA-LUAD with mEGFR (LUAD cohort), which are publicly available, were analyzed and used to verify the mutational characteristics of study cohort. The characteristics of the genetic variations were analyzed in the context of the mutational signature proposed by Alexandrov et al. [17].

## Methods

### Study cases

A total of 74 lung adenocarcinoma tissues which met the following criteria were randomly selected from the tissue archives of two affiliated hospitals, Severance Hospital and Gangnam Severance Hospital, of Yonsei Medical Center (study cohort). Each specimen (1) is a pathologically confirmed lung adenocarcinoma, (2) had history of curative surgical resection, (3) has a submission of informed consent for sequencing of major cancer related genes, (4) was confirmed to have mEGFR either by Sanger sequencing or PNA clamping methods, and (5) has submission of informed consent for tissue collection. This study was approved by the IRB of Gangnam Severance Hospital (IRB #3–2017-0059) and was carried out in compliance with the Declaration of Helsinki (https://www.wma.net/policies-post/wma-declaration-of-helsinki-ethical-principles-for-medical-research-involving-human-subjects/) and Korean GCP guidelines. In order to further evaluate the mutational characteristics of lung adenocarcinoma with mEGFR, publicly available data were extracted from the GDC Data Portal of The Cancer Genome Atlas (TCGA https://portal.gdc.cancer.gov/). Among the 585 TCGA-LUAD cases, 81 cases harbor mutations in the EGFR gene (ENSG00000146648) with 47 types listed in TCGA-LUAD (Additional file 1: Table S1). A total of 7577 mutations were found in 48 TCGA-LUAD cases; TCGA-55-8506 has 2305 mutations alone, accounting for 30.4% of all mutations, and was excluded from further analysis. For this analysis, we selected 47 cases with 7 EGFR subtypes known as common mEGFR (LUAD cohort) (Additional file 2: Table S2 and Additional file 3: Table S3) (8).

**Next generation sequencing**: A 0.62 Mb customized NGS panel containing 70 major cancer genes was constructed and sequenced using the Ion S5 NGS system (Thermo Fisher Scientific) (Additional file 4: Table S4). For the extraction of cancer-enriched gDNA, paraffin-embedded tissue samples were loaded onto silanated slides in 4-μm-thickness sections. Each slide was lightly stained with H&E and examined for the presence of cancer cells. The cancer-enriched area as determined by an independent lung pathologist was marked and scraped with clean blades. gDNA was extracted using a QIAamp DNA FFPE Tissue Kit (Qiagen, Valencia, CA, USA). Fifty ng of extracted DNA was reacted with fragmentizer for 12 to 50 min (Archer) and the section of cut DNA was blunted and 5′ phosphorylated with an end-repair enzyme (Archer). The end of the DNA was barcode-ligated through a reaction with an MBC adapter (Archer) for 15 min, and 1st and 2nd PCR were performed using a primer set for the selected target genes. After measuring the prepared library with Qubit^Ⓡ^, 50 pmol of sample was obtained and mixed with mineral oil to normalize it with beads. The sample was applied to a 540 Chip (Life Technology) and sequenced with the S5 sequencer (Life technology). The obtained results were analyzed by Archer Analysis 5.1 (Archer) and the median depth of the sequencing was 308X [164.5 ~ 738.5].

### Statistics

Categorical and continuous variables were compared using χ^2^-tests and *t*-test, respectively. Differences in the distribution of continuous variables between the two independent samples and dependent samples were assessed by Mann–Whitney U test and Wilcoxon signed-rank test, respectively. The Kruskal-Wallis test was used to compare the medians of three or more groups, and the Bonferroni method was used for the post-hoc test. The mutational signature was analyzed using R software and the Maftools package [18]. Briefly, the pattern of the mutation is analyzed under the 3-nucleotide context, which includes one nucleotide immediately 5′ and 3′ of the mutated base [10, 17] to characterize single base substitution using a 96-mutation classification by combining 6 substitution types and the 5′ and 3′ adjacent bases to the mutated base. The mutation patterns found in cancers are analyzed by non-negative matrix factorization (NMF), and compared to 30 types of mutational signatures [8]. All analyses were performed using SPSS version 23 (SPSS Inc., Chicago, IL, USA). All statistical tests were two-sided, and a P-value < 0.05 was considered to be statistically significant.

## Results

### Characteristics of study cohort

The incidence of lung adenocarcinoma harboring EGFR-TKI-sensitizing mutations is higher in East Asia than in Western countries, and the proportion of non-smokers and women is high, suggesting the presence of mutagenic stressors other than cigarette smoking. We conducted targeted sequencing of 70 major cancer-related genes on a total 73 specimens recruited from 71 patients, including two cases with double primary cancer (Table 1 and Additional file 5: Table S5). These included 37 cases with EGFR exon19 deletion mutation, 35 cases with L858R mutation and one case with the L861Q mutation. The mean age of the patients was 60.34 ± 10.01 years, and they were comprised of 25 males and 46 females. Of these, 20 cases had a history of smoking, with a mean of 22.75 ± 13.33 pack-years.

**Table 1:** Demographic characteristics of the 2 cohorts according to the EGFR-TKI mutation subtypes

			SNV^*^	E19del^**^	P-value
Study cohort (*n* = 71)^†^	Age		62.58 ± 9.67	58.03 ± 9.95	0.0547^‡‡^
	Gender	Male	14	11	0.6822^§^
		Female	22	24	
	Smoking history	Ever smoker	11	9	0.8497^§^
		None smoker	25	26	
	Stage	I	22	12	0.1100^§^
		II	5	12	
		III	8	10	
		IV	1	1	
LUAD cohort (*n* = 47) ^§§^	Age		66.56 ± 9.18	65.81 ± 10.40	0.8138^‡‡^
	Gender	Male	7	4	0.9234^§^
		Female	10	14	
	Smoking history	Ever smoker	15	4	0.0540^§^
		None smoker	13	15	
	Stage	I	13	7	0.9715^§^
		II	7	5	
		III	6	5	
		IV	1	1	

^*^ Study cohort of SNV subgroup includes one L861Q variant and LUAD cohort of SNV subgroup includes 3 cases of L861Q, 2 G719A, and 1 G719C variant

^**^Includes E19 inframe del variants including LREA motif

^†^ Includes 2 cases with double primary tumor

^‡‡^ P-values were obtained by independent sample t-test

^§^ P-values were obtained by chi-square test

^§§^The clinical information of 2 cases is not available

### C > A transversion accounts for small fraction in study cohort

**Fig. 1:**
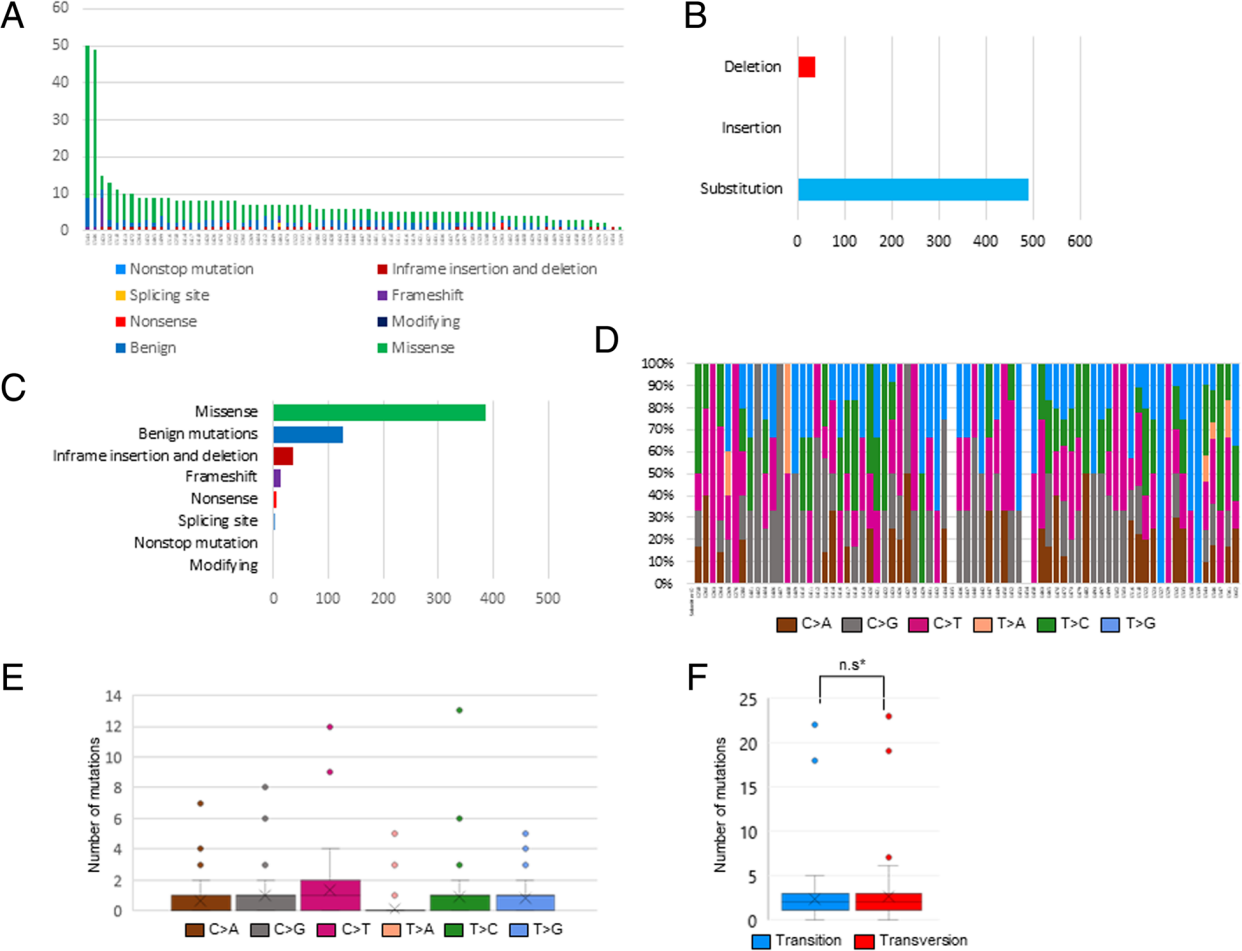
Characteristics of the variations in the study cohort. Histogram showing the cumulative frequency of variation for individual cases (**a**). Stacked bar chart summarizing the variant types of all cases with substitutions, insertions, and deletions (**b**). Bundled bar chart classifying variations using the VEP (**c**). Bundled column chart showing the SNV classification of individual cases (**d**). Box plot summarizing the SNV of study cohort (**e**). Box plot created by dividing the SNV into Ti and Tv (**f**). n.s* not significant

The sequencing of 73 specimens revealed 530 mutations consisting of 490 single and double nucleotide substitutions, 1 insertion, and 39 deletions. The median of total mutations obtained from each sample was 6 [5 ~ 8] (Fig. 1A and B). These mutations were further classified according to the variant effect predictor (VEP) and the results are shown in Fig. 1C and Table 2. C > A transversion, which is attributed to cigarette smoking, is the most common type of SNV in lung cancer [5]; therefore, we questioned whether this finding also applies to Korean lung adenocarcinomas with mEGFR. Excluding the mutations which were assumed to be benign because they did not change protein behaviors by the VEP, and indels and doublets, a total of 363 SNVs were classified and analyzed (Fig. 1D and E). In this subset, C > T transition accounted for the largest portion (27.3%), followed by C > G transition (20.4%), whereas C > A transversion occupied the second smallest fraction (12.9%). The frequency of C > A transversion was statistically significantly lower than those of other mutations, except for T > A and T > G transversions, which differs from the general notion that NSCLC harbors higher C > A transversions due to cigarette smoking. The differences in the frequencies of Ti and Tv did not reach statistically significant level in each individual case (Fig. 1F).

**Table 2:** Classification of variants in study cohort according to the VEP

Type of variants	Number of mutations
Missense	346
Benign mutations	127
Modifying	0
Nonsense	6
Frameshift	13
Splicing site	1
Inframe insertion and deletion	37
Nonstop mutation	0
Total	530

### Lung adenocarcinoma with the E19del mutation has a significantly lower mutational burden, higher Ti/Tv ratio

**Fig. 2:**
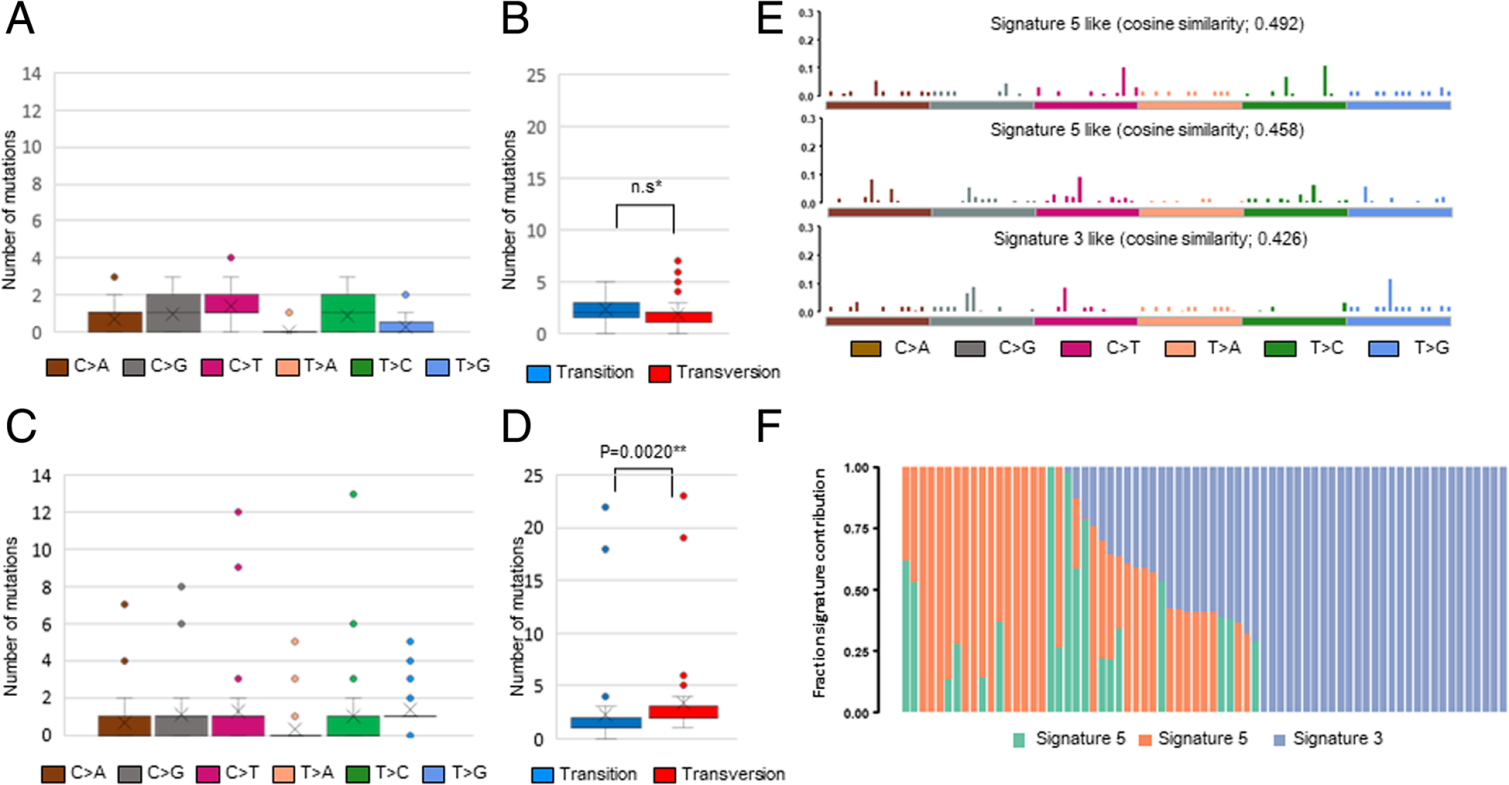
Mutational pattern of study cohort. Box plot summarizing the SNV and box plot of Ti and Tv in the E19del-subgroup (**a** and **b**) and in the SNV-subgroup (**c** and **d**). Mutational signature plot derived from a 74 X 96 matrix analyzed using the NMF, where 74 is the number of the study cohort (**e**) and bar graph indicating the degree to which each case contributed to the signature (**f**). ^*^n.s. not significant. ** P-value was obtained by Wilcoxon signed rank test

To identify the presence of smoking effects, we examined whether there was a difference in the mutational spectrum between cases with vs. without a smoking history. There was no statistically significant difference in the total number of mutations between smoker and non-smoker groups and between those with more than a 20-pack-year history and the other cases. There was no significant difference in the frequencies of Ti, Tv, and Ti/Tv ratio according to the smoking history. These findings were similar to the comparison of mutation patterns according to gender, which showed no differences in the total mutational burden, frequencies of Ti and Tv, and Ti/Tv ratios between genders. Then, we questioned whether the subtypes of mEGFR are related to the difference in mutation burden, Ti, Tv, and Ti/Tv ratio. In order to accomplish this, the cases were classified into either the mEGFR E19del-subgroup or the mEGFR SNV-subgroup, which includes cases with L858R, L861Q, and other EGFR-TKI sensitizing SNVs. In the E19del-subgroup, there was no statistically significant difference in the frequency of Ti and Tv in each individual, whereas the frequency of Tv was significantly higher than Ti in the mEGFR SNV-subgroup (*P* = 0.0020, Wilcoxon signed rank test). There was a significant difference in the total number of mutations, as the E19del-subgroup had a lower mutational burden than the SNV-subgroup (*P* = 0.0326, Mann-Whitney U test). The E19del-subgroup had lower Tv and a higher Ti and Ti/Tv ratio than the SNV-subgroup (*P* = 0.0441, =0.0020, and = 0.0002, respectively, Mann-Whitney U test, Fig. 2A~D). As C > A transversion was less frequent than in general lung cancer, this suggests that some mutagenic stress other than cigarette smoking likely exists in this subset of lung adenocarcinomas. To identify the mutagenic stressors that influence mEGFR-positive lung adenocarcinomas, we analyzed the mutational signature proposed by Alexandrov et al. (4). When the three best-matched mutational signatures were estimated using non-negative matrix factoralization (NMF), signature 5 and signature 3 was derived (Fig. 2E and F). Among smoking history, gender, and EGFR-mutation subtype, only EGFR subtype showed a difference in the distributions of Ti and Tv. We further analyzed the mutational signatures between the E19del-subgroup and the SNV-subgroup using NMF. In the subgroup analysis on the mutational, signature 5 is the predominant mutational signature in this study cohort (Data not shown).

### LUAD cohort has a lower mutational burden than other TCGA cancer cohorts

**Fig. 3:**
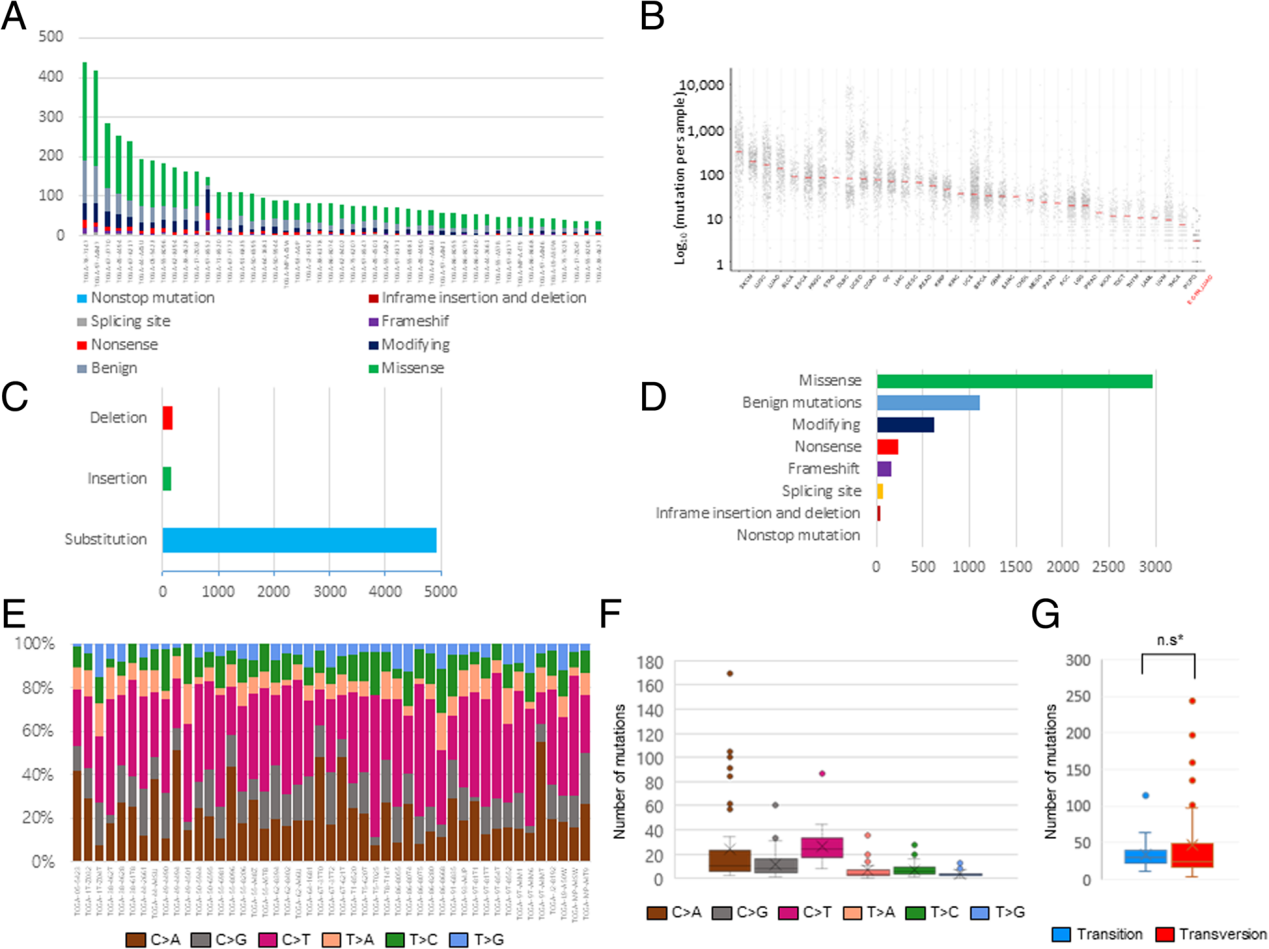
Characterization of mutations in LUAD cohort. Histogram showing the cumulative frequency of variation for individual cases (**a**). Scatter plot comparing the mutational loads of LUAD cohort with other TCGA cancer cohorts (**b**). Stacked bar chart summarizing the variant types of all cases with substitutions, insertions, and deletions (**c**). Bundled bar chart classifying variations using the VEP (**d**). Bundled column chart showing the SNV classification of individual cases (**e**). Box plot summarizing the SNV of all cases (**f**) Box. Plot created by dividing the SNV into Ti and Tv (**g**). ^*^n.s. not significant

The mean age of the 47 selected cases was 66.20 ± 9.44 years, and the cases were comprised of 11 males, 34 females, and two cases in which the gender was not reported (Table 1). Of these, 19 had a smoking history. A total of 5272 mutations were recovered from 47 EGFR-TKI-sensitive mutation-positive TCGA-LUAD cases. The median number of total mutations obtained from each sample was 78 [56 ~ 112.5], which indicates that the mutational burden was significantly lower than that of other cancer types listed in the TCGA dataset and the TCGA-LUAD parent group (Fig. 3A and B). These 5272 mutations are classified into 4954 single nucleotide substitutions, 146 insertions, and 172 deletions (Fig. 3C). These mutations were further classified according to the VEP and the results are shown in the Table 3 and Fig. 3D.

**Table 3:** Classification of variants in LUAD cohort according to the VEP

Type of variant	Number of mutations
Missense	2986
Benign mutations	1127
Modifying	629
Nonsense	243
Frameshift	163
Splicing site	74
Inframe insertion and deletion	48
Nonstop mutation	2
Total	5272

### C > T transition is the most common type of SNV

Because C > A transversion, which is attributed to cigarette smoking, is the most common variation in NSCLC [5], we questioned whether this finding also applies to this subset of NSCLC. 1179 mutations, including 1071 synonymous mutations, 47 mutations in the splice region, and 1 stop retaining mutation, were assumed to be harmless or unlikely to change protein behaviors by the VEP; these SNVs along with insertions and deletions were excluded, and 3812 SNVs were classified and analyzed (Fig. 3E and F). In this subset, C > T transition accounted for the largest portion (32.7%), followed by C > A transversion (30.3%). There was no significant difference in the frequency of C > T vs. C > A mutations in each individual, which contradicts the general notion that NSCLCs harbor higher C > A transversions than any other SNV. The difference in the frequencies of Ti and Tv in each individual case was not statistically significant (Fig. 3G).

### Different mutational spectra between the E19del and SNV-subgroups

**Fig. 4:**
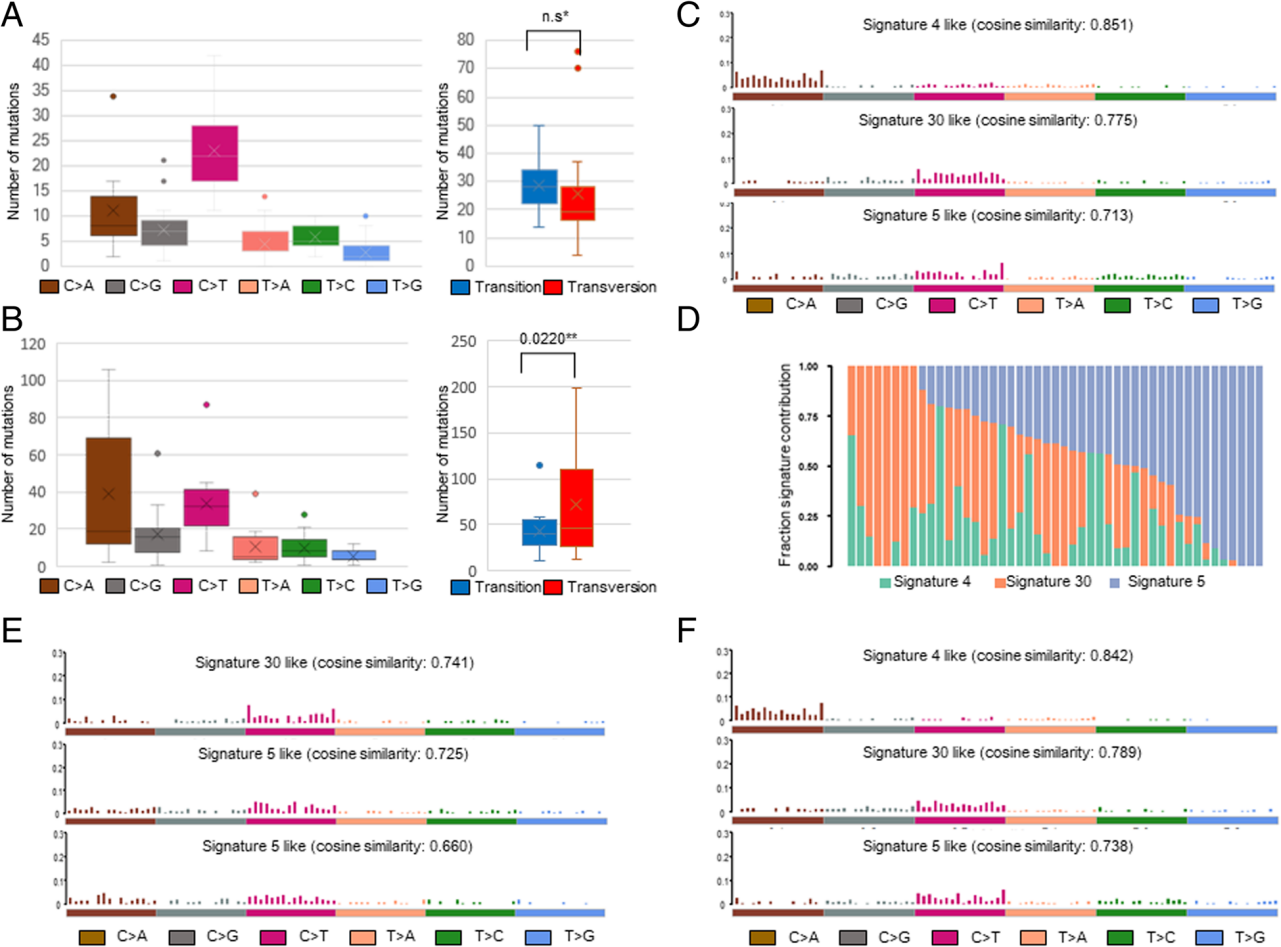
Mutational signatures of LUAD cohort. Box plot summarizing the SNV and box plot of Ti and Tv in the LUAD E19del-subgroup (**a**) and in the LUAD SNV-subgroup (**b**). Mutational signature plot derived from a 47 X 96 matrix analyzed using the NMF, which had 37 mEGFR -positive TCGA-LUAD cases (**c**) and bar graph indicating the degree to which each case contributed to the individual mutational signature (**d**). Mutational signatures obtained from the E19del- subgroup (**e**) and LUAD SNV-subgroup (**f**). ^*^n.s. not significant. ** P-value was obtained by Wilcoxon signed rank test

The differences in the mutational pattern observed in the study cohort were further verified in the LUAD cohort. The mutational burden did not differ between the groups with and without a smoking history. In addition, there were no differences in the Ti, Tv, and Ti/Tv ratio according to smoking history or between smokers and non-smokers. There was also no significant difference in the total mutational burden or the frequency of Ti, Tv, and Ti/Tv ratio between genders. Finally, we questioned whether the subtypes of EGFR-TKI-sensitizing mutations were related to the difference in mutational burden, Ti, Tv, and Ti/Tv ratio. As seen in the study cohort, there was no statistically significant difference in the frequency of Ti and Tv in each individual in the E19del-subgroup, whereas the frequency of Tv was significantly higher than Ti in the mEGFR SNV-subgroup (*P* = 0.0220, Wilcoxon signed rank test). In the E19del-subgroup, the total mutation burden and the number of Tv mutations were significantly lower than in the SNV-subgroup (*p* = 0.0497 and = 0.0220, respectively, Mann-Whitney U test) and the Ti/Tv ratio was significantly higher as well (*P* = 0.0055, Mann-Whitney U test) (Fig. 4A and B).

### Heterogeneity of mutational signatures between TCGA-LUAD cases with EGFR-TKI-sensitizing mutations

Next, in order to estimate the mutagenic stress that influenced EGFR mutations, we analyzed the LUAD cohort using the mutational signature proposed by Alexandrov et al. (4). When inferring the mutagenic stress using NMF, signature 4 (cosine similarity = 0.851), signature 30 (cosine similarity = 0.775), and signature 5 (cosine similarity = 0.713) were derived (Fig. 4C and D). Because the subset analysis of LUAD cohort according to smoking history, gender, and EGFR mutation subtype showed that only the subtype influenced the distribution of mutational burden, Ti, and Tv, we compared the mutational signatures between the E19del-subgroup and the SNV-subgroup. In the E19del-subgroup, signature 30 (cosine similarity = 0.741, unknown etiology) and signature 5 (cosine similarity = 0.725 and = 0.660, unknown etiology) were derived, whereas signature 4 (cosine similarity = 0.842, smoking effect), signature 30 (cosine similarity = 0.789, unknown etiology) and signature 5 (cosine similarity = 0.738 unknown etiology) were obtained from the SNV-subgroup (Fig. 4E and F). Taken together, the mutational signatures differ between the E19del-subgroup and the SNV-subgroup, as smoking related signature 4 was observed only in SNV-subgroup, and signature 5 and 30 are the prevalent underlying mutational signature in lung adenocarcinoma with mEGFR-TKI.

## Discussion

mEGFR-positive lung adenocarcinoma is a distinctive subtype of lung cancer which attracts attention because it is a prevalent disease which accounts for about half of East Asian lung adenocarcinomas and because of the facts that it does not involve the typical risk factors for lung cancer such as age, gender, and smoking [19].

The proportion of C > A transversions, which is related to tobacco smoking, was not the major SNV, whereas C > T transitions comprise the highest proportions of mutations in both cohorts. Another interesting finding is that the proportion of C > A transversions is relatively high in the LUAD cohort than study cohort, which may be explained by the fact that the cases in the LUAD cohort had a higher smoking history than that of the study cohort (LAUD cohort: 40.4% vs. study cohort: 27.8%). Further analysis of LUAD cohort according to EGFR subtype revealed that the Tv frequency was relatively higher, and mutation signature 4 was observed in the SNV-subgroup. Although signature 4 was not derived in the study cohort, it is presumed that mutagenic stressors such as smoking are related to the L858R mutation because the Tv frequency is higher, similar to the LUAD cohort. The E19del-subgroup had lower mutational burden than the SNV subgroup. Both the data from the targeted panel of this study and whole exome data from TCGA-LUAD showed that the mutations of E19del subgroup randomly distributed throughout the genome and the obvious causes could not be detected in the demographic characteristics of E19del subgroup. Younger age in the study cohort and less smoking history in LUAD cohort subgroup might be attributed to these findings. Mutational signature 5 was main variation pattern in the study cohort, whereas signatures 4, 30, and 5 were derived from the analysis of the LUAD cohort. These differences may be attributed to factors such as the higher proportion of smokers in the EGFR-L858R group and higher age in the LUAD cohort compared to the study cohort (LUAD cohort: 66.30 ± 9.54 vs. study cohort: 60.51 ± 10.31 years), and unidentified racial differences. However, during additional analysis by subgroups, signature 5 was predominant and commonly derived, indicating that this maybe one of the key mutational signatures in this cancer type.

The underlying mutational mechanism for signature 5, which exhibits a transcriptional strand bias for T > C substitutions at ApTpN context, is yet to be well elucidated. This signature is common in papillary cell renal carcinoma, neuroblastoma, and clear cell renal carcinoma and, in some cancer types, is associated with increased age. However, the correlation between signature 5 and increased age was not observed in the analysis of whole exome sequencing of lung cancer, and it was observed even when we examined the demographic characteristics of our lung cancer set [20]. Among the cancers arising in the kidney, this mutation is characteristic of clear cell and papillary renal cell carcinoma, which absorbs metabolites continuously, whereas it is low in chromophobe renal carcinoma in cortical collecting ducts, suggesting that it may be attributable to the replication error of deaminated cytosine and adenine [21]. Indeed, relentless efforts are required to find out mutagenic stressors other than smoking, such as radon, indoor emissions from household combustion, and exhaust from diesel engines, by collecting the cases enriched with signature 5 and investigating them in various aspects. In contrast, the mutational signature 30, found in a small subset of breast cancers, was observed in the analysis of LUAD cohort; the cause of this mutation pattern has yet to be estimated.

To find out recurrent mutations in specific genes according to the mEGFR subtypes, concurrent mutation was detected by oncoplot and then detected mutations were further examined using the oncodrive function in maftools package, which based on algorithm oncodriveCLUST [22]. In this inspection, the E19del subgroup of study cohort had concurrent mutations in the following order; TP53 > IDH2 > FBXW7 and in the SNV subgroup; TP53 > FBXW7 > KRAS. On the other hands, E19del subgroup of LUAD cohort has concurrent mutations as following order; CDKN2A > CEP76 > KIAA2026 and SNV subgroup; AP3D1 > EMR1 > FASTKD3. The mutations observed here were randomly distributed on the genes, and other recurrent driver mutations except mEGFR were not derived.

Targeted sequencing using the Foundation One panel could reflect the results of whole exome sequencing, in terms of the mutational burden [23]. This study was carried out based on this assumption, however, the analysis of study cohort using a customized panel containing 70 major genes covering 0.62 Mb is concerning in terms of direct comparison with the LUAD cohort, which is based on whole exome sequencing. In the future, if the cost is further reduced, it may be necessary to find a minimum sequencing area that can represent whole exome sequencing.

Taken together, the subtype of lung adenocarcinoma with EGFR-TKI-sensitizing mutations does not show a characteristic mutation pattern influenced by smoking and additionally shows a low incidence of C > A transversion, which is a common feature of lung cancer; it also had a mutational burden lower than those of other TCGA cancers. E19del and L858R, which are representative subtypes of lung adenocarcinoma, differ in the characteristics of mutations, as the E19del group has a lower mutation burden and a higher ratio of transition than the transversion mutations. Overall, the presence of mutational signatures 5 and 30 was a predominant pattern observed across the subtypes, but the main factors related to this type of signature are still unknown, so they require further in-depth studies on signature 5 and 30 in this particular subtype of lung cancer.

## Conclusions

Lung adenocarcinoma with mEGFR(+) has a lower mutational burden and does not show a characteristic mutation pattern influenced by smoking. E19del and L858R, which are representative subtypes of mEGFR(+) lung adenocarcinoma, differ in terms of mutational spectra, as the E19del group has a lower mutation burden and a higher Ti/Tv ratio. These findings could explain on the differences in the responses to EGFR-TKIs and clinical courses between the two lung adenocarcinoma subgroups.

## Additional files

Additional file 1: **Table S1.** Type of EGFR mutations in TCGA-LUAD. (DOCX 15 kb)

Additional file 2: **Table S2.** List of mEGFR included in this study. (DOCX 12 kb)

Additional file 3: **Table S3.** List of TCGA-LUAD cases recruited for this study (LUAD cohort). (DOCX 14 kb)

Additional file 4: **Table S4.** List of genes included in the customized NGS panel. (DOCX 12 kb)

Additional file 5: **Table S5.** List of study cohort recruited for this study (study cohort). (DOCX 17 kb)

## Abbreviations

EGFR: *Epidermal growth factor receptor*
*mEGFR*: *EGFR-TKI sensitizing mutation*
*NGS*: Next-generation sequencing
NSCLC: non-small cell lung cancer
SNV: single nucleotide variation
Ti: transition
TKI: tyrosine kinase inhibitor
Tv: transversion
VEP: variant effect predictor
