# Supplementary material for: Different mutational characteristics of the subsets of EGFR-tyrosine kinase inhibitor sensitizing mutation-positive lung adenocarcinoma
Source: BMC Cancer. 2018 Dec 6;18:1221. doi: 10.1186/s12885-018-5116-9 (PMC6282318; doi:10.1186/s12885-018-5116-9)
Supplement: Supplementary file 1 — Table S1. Type of EGFR mutations in TCGA-LUAD. (DOCX 15 kb) [file 12885_2018_5116_MOESM1_ESM.docx]

Supplementary Table 1. Type of EGFR mutations in TCGA-LUAD.

| No | DNA Change | Type | Consequences | # Affected Cases in TCGA-LUAD Cohort (n=81, (%)) | Impact (VEP) |
| --- | --- | --- | --- | --- | --- |
| 1 | chr7:g.55191822T>G | Substitution | Missense EGFR L858R | 23 (0.284) | MODERATE |
| 2 | chr7:g.55174772delGGAATTAA… | Deletion | Inframe Deletion EGFR E746_A750del | 12 (0.1481) | MODERATE |
| 3 | chr7:g.55174773delGAATTAAG… | Deletion | Inframe Deletion EGFR E746_A750del | 5 (0.0617) | MODERATE |
| 4 | chr7:g.55191831T>A | Substitution | Missense EGFR L861Q | 3 (0.037) | MODERATE |
| 5 | chr7:g.55174792C>T | Substitution | Missense EGFR S752F | 3 (0.037) | MODERATE |
| 6 | chr7:g.55173986delAAC | Deletion | Inframe Deletion EGFR E709_T710... | 3 (0.037) | MODERATE |
| 7 | chr7:g.55174015G>C | Substitution | Missense EGFR G719A | 2 (0.0247) | MODERATE |
| 8 | chr7:g.55174775delATTAAGAG… | Deletion | Inframe Deletion EGFR L747_T751del | 2 (0.0247) | MODERATE |
| 9 | chr7:g.55174785G>C | Substitution | Missense EGFR A750P | 2 (0.0247) | MODERATE |
| 10 | chr7:g.55181378C>T | Substitution | Missense EGFR T790M | 2 (0.0247) | MODERATE |
| 11 | chr7:g.55142382T>G | Substitution | Missense EGFR L62R | 2 (0.0247) | MODERATE |
| 12 | chr7:g.55181312G>T | Substitution | Missense EGFR S768I | 2 (0.0247) | MODERATE |
| 13 | chr7:g.55143335A>G | Substitution | Missense EGFR I91V | 1 (0.0123) | MODERATE |
| 14 | chr7:g.55156657G>T | Substitution | Missense EGFR R377S | 1 (0.0123) | MODERATE |
| 15 | chr7:g.55201775G>T | Substitution | Missense EGFR R1052I | 1 (0.0123) | MODERATE |
| 16 | chr7:g.55205666A>T | Substitution | 3 Prime UTR EGFR | 1 (0.0123) | MODIFIER |
| 17 | chr7:g.55174021G>T | Substitution | Missense EGFR G721V | 1 (0.0123) | MODERATE |
| 18 | chr7:g.55174798A>T | Substitution | Missense EGFR K754I | 1 (0.0123) | MODERATE |
| 19 | chr7:g.55198778C>A | Substitution | Missense EGFR S921R | 1 (0.0123) | MODERATE |
| 20 | chr7:g.55198717G>T | Substitution | Missense EGFR G901V | 1 (0.0123) | MODERATE |
| 21 | chr7:g.55181309_55181310insCAGCGTGG… | Insertion | Inframe Insertion EGFR S768_D770du | 1 (0.0123) | MODERATE |
| 22 | chr7:g.55174788A>C | Substitution | Missense EGFR T751P | 1 (0.0123) | MODERATE |
| 23 | chr7:g.55160233G>A | Substitution | Missense EGFR G465R | 1 (0.0123) | MODERATE |
| 24 | chr7:g.55157751A>T | Substitution | Missense EGFR Q432H | 1 (0.0123) | MODERATE |
| 25 | chr7:g.55163734G>C | Substitution | Missense EGFR E545Q | 1 (0.0123) | MODERATE |
| 26 | chr7:g.55161608G>T | Substitution | Synonymous EGFR V536V | 1 (0.0123) | LOW |
| 27 | chr7:g.55174795C>A | Substitution | Missense EGFR P753Q | 1 (0.0123) | MODERATE |
| 28 | chr7:g.55191748G>T | Substitution | Missense EGFR L833F | 1 (0.0123) | MODERATE |
| 29 | chr7:g.55181305_55181306insTGGCCAGC… | Insertion | Inframe Insertion EGFR A767_V769du | 1 (0.0123) | MODERATE |
| 30 | chr7:g.55191746T>G | Substitution | Missense EGFR L833V | 1 (0.0123) | MODERATE |
| 31 | chr7:g.55156784C>A | Substitution | Missense EGFR L387M | 1 (0.0123) | MODERATE |
| 32 | chr7:g.55155838G>A | Substitution | Missense EGFR V300M | 1 (0.0123) | MODERATE |
| 33 | chr7:g.55174796G>T | Substitution | Synonymous EGFR P753P | 1 (0.0123) | LOW |
| 34 | chr7:g.55160296C>T | Substitution | Stop Gained EGFR Q486* | 1 (0.0123) | HIGH |
| 35 | chr7:g.55174014G>T | Substitution | Missense EGFR G719C | 1 (0.0123) | MODERATE |
| 36 | chr7:g.55152582G>T | Substitution | Missense EGFR R222L | 1 (0.0123) | MODERATE |
| 37 | chr7:g.55174797delAAAGCCAA… | Deletion | Inframe Deletion EGFR K754_I759del | 1 (0.0123) | MODERATE |
| 38 | chr7:g.55152547G>T | Substitution | Splice Region EGFR L210L | 1 (0.0123) | LOW |
| 39 | chr7:g.55202603delC | Deletion | Frameshift EGFR D1083Efs*11 | 1 (0.0123) | HIGH |
| 40 | chr7:g.55191845G>A | Substitution | Missense EGFR E866K | 1 (0.0123) | MODERATE |
| 41 | chr7:g.55174791delTCTCCGAA… | Deletion | Frameshift EGFR S752Pfs*3 | 1 (0.0123) | HIGH |
| 42 | chr7:g.55174789delC | Deletion | Frameshift EGFR T751Nfs*15 | 1 (0.0123) | HIGH |
| 43 | chr7:g.55181319_55181320insGGGTTA | Insertion | Inframe Insertion EGFR D770_N771... | 1 (0.0123) | MODERATE |
| 44 | chr7:g.55174797A>G | Substitution | Missense EGFR K754E | 1 (0.0123) | MODERATE |
| 45 | chr7:g.55191808C>T | Substitution | Synonymous EGFR I853I | 1 (0.0123) | LOW |
| 46 | chr7:g.55198734T>A | Substitution | Missense EGFR L907M | 1 (0.0123) | MODERATE |
| 47 | chr7:g.55173019G>T | Substitution | Synonymous EGFR G652G | 1 (0.0123) | LOW |
