# Supplementary material for: Different mutational characteristics of the subsets of EGFR-tyrosine kinase inhibitor sensitizing mutation-positive lung adenocarcinoma
Source: BMC Cancer. 2018 Dec 6;18:1221. doi: 10.1186/s12885-018-5116-9 (PMC6282318; doi:10.1186/s12885-018-5116-9)
Supplement: Supplementary file 2 — Table S2. List of mEGFR included in this study. (DOCX 12 kb) [file 12885_2018_5116_MOESM2_ESM.docx]

Supplementary table 2. List of mEGFR included in this study.

|  | DNA Change | Type | Consequences |
| --- | --- | --- | --- |
| 1 | chr7:g.55191822T>G | Substitution | Missense EGFR L858R |
| 2 | chr7:g.55174772delGGAATTAA… | Deletion | Inframe Deletion EGFR E746_A750del |
| 3 | chr7:g.55174773delGAATTAAG… | Deletion | Inframe Deletion EGFR E746_A750del |
| 4 | chr7:g.55191831T>A | Substitution | Missense EGFR L861Q |
| 5 | chr7:g.55174015G>C | Substitution | Missense EGFR G719A |
| 6 | chr7:g.55174775delATTAAGAG… | Deletion | Inframe Deletion EGFR L747_T751del |
| 7 | chr7:g.55174014G>T | Substitution | Missense EGFR G719C |
