# Supplementary material for: Different mutational characteristics of the subsets of EGFR-tyrosine kinase inhibitor sensitizing mutation-positive lung adenocarcinoma
Source: BMC Cancer. 2018 Dec 6;18:1221. doi: 10.1186/s12885-018-5116-9 (PMC6282318; doi:10.1186/s12885-018-5116-9)
Supplement: Supplementary file 3 — Table S3. List of TCGA-LUAD cases recruited for this study (LUAD cohort). (DOCX 14 kb) [file 12885_2018_5116_MOESM3_ESM.docx]

Supplementary Table 3. List of TCGA-LUAD cases recruited for this study (LUAD cohort).

| Number | Submitter ID | Consequences | project/project_id | demographic/race | demographic/gender | Smoking history | Tumour Stage |
| --- | --- | --- | --- | --- | --- | --- | --- |
| 1 | TCGA-67-6217 | L858R | TCGA-LUAD | white | female | no | iia |
| 2 | TCGA-MP-A4SW | L858R | TCGA-LUAD | white | male | yes | iib |
| 3 | TCGA-67-3772 | L858R | TCGA-LUAD | white | female | no | ib |
| 4 | TCGA-62-8394 | L858R | TCGA-LUAD | white | female | no | iiib |
| 5 | TCGA-97-8177 | L858R | TCGA-LUAD | white | female | no | ib |
| 6 | TCGA-86-8055 | L858R | TCGA-LUAD | white | male | yes | iia |
| 7 | TCGA-49-4490 | L858R | TCGA-LUAD | white | female | yes | iiia |
| 8 | TCGA-67-3770 | L858R | TCGA-LUAD | white | female | no | ia |
| 9 | TCGA-MP-A4T9 | L858R | TCGA-LUAD | white | female | yes | iiia |
| 10 | TCGA-97-A4M7 | L858R | TCGA-LUAD | white | male | yes | ia |
| 11 | TCGA-L9-A50W | L858R | TCGA-LUAD | black or african american | male | yes | iia |
| 12 | TCGA-64-1681 | L858R | TCGA-LUAD | white | female | yes | ia |
| 13 | TCGA-86-8075 | L858R | TCGA-LUAD | white | female | no | ib |
| 14 | TCGA-38-4627 | L858R | TCGA-LUAD | white | female | no | iia |
| 15 | TCGA-97-A4M1 | L858R | TCGA-LUAD | white | female | yes | ia |
| 16 | TCGA-17-Z047 | L858R | TCGA-LUAD | N.A | N.A | N.A | N.A |
| 17 | TCGA-50-5944 | L858R | TCGA-LUAD | white | female | no | ia |
| 18 | TCGA-86-8668 | L858R | TCGA-LUAD | white | female | no | ia |
| 19 | TCGA-49-4494 | L858R | TCGA-LUAD | white | male | yes | iiia |
| 20 | TCGA-71-8520 | L858R | TCGA-LUAD | asian | female | no | ib |
| 21 | TCGA-55-8096 | L858R | TCGA-LUAD | white | female | yes | ib |
| 22 | TCGA-91-6835 | L858R | TCGA-LUAD | white | female | yes | ia |
| 23 | TCGA-17-Z032 | E19del | TCGA-LUAD | N.A | N.A | N.A | N.A |
| 24 | TCGA-75-6207 | E19del | TCGA-LUAD | not reported | male | yes | iiia |
| 25 | TCGA-97-8171 | E19del | TCGA-LUAD | asian | male | yes | iv |
| 26 | TCGA-38-6178 | E19del | TCGA-LUAD | white | female | no | iiia |
| 27 | TCGA-97-A4M6 | E19del | TCGA-LUAD | white | female | no | ia |
| 28 | TCGA-86-8280 | E19del | TCGA-LUAD | white | female | no | iia |
| 29 | TCGA-38-4628 | E19del | TCGA-LUAD | white | female | no | iib |
| 30 | TCGA-75-7025 | E19del | TCGA-LUAD | not reported | male | yes | ib |
| 31 | TCGA-44-2661 | E19del | TCGA-LUAD | white | female | no | ia |
| 32 | TCGA-86-8074 | E19del | TCGA-LUAD | white | female | yes | iia |
| 33 | TCGA-97-8547 | E19del | TCGA-LUAD | not reported | female | no | iiia |
| 34 | TCGA-62-8402 | E19del | TCGA-LUAD | white | female | no | iiia |
| 35 | TCGA-62-A46U | E19del | TCGA-LUAD | white | female | no | iib |
| 36 | TCGA-49-4501 | E19del | TCGA-LUAD | white | female | no | ib |
| 37 | TCGA-97-8552 | E19del | TCGA-LUAD | white | female | no | i |
| 38 | TCGA-J2-8192 | E19del | TCGA-LUAD | white | female | no | iia |
| 39 | TCGA-55-8206 | E19del | TCGA-LUAD | white | male | no | ia |
| 40 | TCGA-93-A4JP | L861Q | TCGA-LUAD | asian | male | no | iv |
| 41 | TCGA-05-5423 | L861Q | TCGA-LUAD | not reported | male | yes | iib |
| 42 | TCGA-78-7147 | L861Q | TCGA-LUAD | white | female | yes | iib |
| 43 | TCGA-50-6595 | G719A | TCGA-LUAD | white | female | no | iiia |
| 44 | TCGA-55-A48Z | G719A | TCGA-LUAD | white | female | yes | iiib |
| 45 | TCGA-55-A57B | E19del | TCGA-LUAD | black or african american | female | no | ia |
| 46 | TCGA-55-6981 | E19del | TCGA-LUAD | white | female | no | iiia |
| 47 | TCGA-44-A4SU | G719C | TCGA-LUAD | white | female | yes | ia |
