# Supplementary material for: Different mutational characteristics of the subsets of EGFR-tyrosine kinase inhibitor sensitizing mutation-positive lung adenocarcinoma
Source: BMC Cancer. 2018 Dec 6;18:1221. doi: 10.1186/s12885-018-5116-9 (PMC6282318; doi:10.1186/s12885-018-5116-9)
Supplement: Supplementary file 4 — Table S4. List of genes included in the customized NGS panel. (DOCX 12 kb) [file 12885_2018_5116_MOESM4_ESM.docx]

Supplementary table 4. List of gene included in the customized NGS panel.

| ERBB3 | GNA11 | KDR | NOTCH1 | RHOA | VHL |
| --- | --- | --- | --- | --- | --- |
| ERBB4 | GNAQ | KIT | NPM1 | RICTOR |  |
| ESR1 | GNAS | KRAS | NRAS | ROS1 |  |
| EZH2 | H3F3A | MAP2K1 | PDGFRA | SMAD4 |  |
| FBXW7 | HNF1A | MDM2 | PIK3CA | SMARCB1 |  |
| FGFR1 | HRAS | MET | PIK3R1 | SMO |  |
| FGFR2 | IDH1 | MLH1 | PTEN | SRC |  |
| FGFR3 | IDH2 | MPL | PTPN11 | STK11 |  |
| FLT3 | JAK2 | MYC | RB1 | TERT |  |
| FOXL2 | JAK3 | MYCN | RET | TP53 |  |
