# Supplementary material for: Different mutational characteristics of the subsets of EGFR-tyrosine kinase inhibitor sensitizing mutation-positive lung adenocarcinoma
Source: BMC Cancer. 2018 Dec 6;18:1221. doi: 10.1186/s12885-018-5116-9 (PMC6282318; doi:10.1186/s12885-018-5116-9)
Supplement: Supplementary file 5 — Table S5. List of study cohort recruited for this study (study cohort). (DOCX 17 kb) [file 12885_2018_5116_MOESM5_ESM.docx]

Supplementary Table 5. List of study cohort recruited for this study (study cohort).

| Number | Submetter ID | Consequences | project/project_id | demographic/race | demographic/gender | Smoking history | Tumor_stage | Tumor diameter (cm) | Specified Pathology |
| --- | --- | --- | --- | --- | --- | --- | --- | --- | --- |
| 1 | E258 | E19del | Study | Korean | male | Yes | iia | 5 | Invasive adenocarcinoma, mixed type(acinar and papillary type) |
| 2 | E260 | E19del | Study | Korean | female | No | iia | 2 | Adenocarcinoma, bronchiolar-alveolar type |
| 3 | E263 | E19del | Study | Korean | male | Yes | iia | 4 | mixed type (predominantly acinar type and solid type), poorly differentiated with extensive necrosis |
| 4 | E264 | E19del | Study | Korean | male | Yes | iiia | 2.8 | mixed type (Papillary type and acinar type) |
| 5 | E269 | L858R | Study | Korean | male | Yes | iiia | 4.3 | acinar type, moderatetly differentiated, in the large segment |
| 6 | E276 | E19del | Study | Korean | female | No | iiia | N.A | Adenocarcinoma, moderately differentiated,papillary type |
| 7 | E280 | L858R | Study | Korean | female | No | iia | 6 | Adenocarcinoma, moderately differentiated (size: 6x5cm), mixed type (acinar, papillary, and solid type), with necrosis |
| 8 | E401 | L858R | Study | Korean | female | No | ia | 1.1 | Invasive adenocarcinoma, acinar predominant, (acinar 85% and lepidic 15%) |
| 9 | E402 | E19del | Study | Korean | female | No | ia | 2.8 | Invasive adenocarcinoma, acinar predominant, (acinar 75%, lepidic 10%, papillary 10% and micropapillary 5%) |
| 10 | E404 | L858R | Study | Korean | male | Yes | ia | 1.3 | Invasive adenocarcinoma, acinar predominant, (acinar 50%, lepidic 40% and papillary 10%) |
| 11 | E406 | L858R | Study | Korean | male | Yes | ia | 2.7 | Invasive adenocarcinoma, acinar predominant (acinar 60% and lepidic 40%) |
| 12 | E407 | E19del | Study | Korean | female | No | ib | 3.5 | Invasive adenocarcinoma, papillary predominant, (papillary 60%, acinar 25%, lepidic 10%, micropapillary 5%) |
| 13 | E408 | L861Q | Study | Korean | female | No | ia | 2.5 | Invasive adenocarcinoma, lepidic predominant, (lepidic 50%, acinar 30% and papillary 20%) |
| 14 | E409 | L858R | Study | Korean | male | Yes | ia | 2.5 | Invasive adenocarcinoma, acinar predominant (acinar 60%, papillary 25%, lepidic 10% and micropapillary 5% ) |
| 15 | E410 | L858R | Study | Korean | female | No | ia | 1.8 | Invasive adenocarcinoma, acinar predominant (acinar 90% and lepidic 10%) |
| 16 | E411 | L858R | Study | Korean | male | No | ia | 1.9 | Invasive adenocarcinoma, papillary predominant, (papillary 45%, acinar 40% (cribriform 20%), solid 10% and micropapillary 5% ) |
| 17 | E412 | E19del | Study | Korean | male | Yes | ia | 1.5 | Invasive adenocarcinoma, lepidic predominant,(lepidic 70% and acinar 30%) |
| 18 | E413 | E19del | Study | Korean | female | Yes | ia | 1.9 | Invasive adenocarcinoma, papillary predominant ( papillary 60%, acinar 15% (cribriform), solid 15%, micropapillary 10%) |
| 19 | E414 | L858R | Study | Korean | male | No | ia | 1.8 | Invasive adenocarcinoma, acinar predominant (acinar 55% (cribrifrom 5%), papillary 35%, micropapillary 5%, lepidic 5%) |
| 20 | E416 | L858R | Study | Korean | female | No | ia | 2.9 | Invasive adenocarcinoma, papillary predominant (papillary 45%, micropapillary 30% and acinar 25%) |
| 21 | E417 | L858R | Study | Korean | female | No | ia | 2.5 | Invasive adenocarcinoma, acinar 50%, solid 40%, micropapillary 10% |
| 22 | E418 | L858R | Study | Korean | male | Yes | ia | 1.2 | Invasive adenocarcinoma, papillary predominant, (papillary 50%, acinar 40%, lepidic 10%) |
| 23 | E419 | L858R | Study | Korean | female | Yes | ia | 2.8 | Invasive adenocarcinoma, acinar predominant (acinar 75%, lepidic 15% and papillary 10%) |
| 24 | E420 | E19del | Study | Korean | female | No | iia | 2.1 | Invasive a0denocarcinoma, solid predominant ( solid 80% and acinar 20% |
| 25 | E421 | L858R | Study | Korean | male | No | ia | 2.6 | Invasive adenocarcinoma, acinar predominant (acinar 60%, lepidic 35%, micropapillary 5%) |
| 26 | E422 | E19del | Study | Korean | female | No | ia | 1.8 | Invasive adenocarcinoma, acinar predominant (acinar 60%, solid 20%, papillary 10%, micropapillary 5%, and lepidic 5%) |
| 27 | E423 | E19del | Study | Korean | male | Yes | ib | 3.2 | invasive adenocarcinoma, acinar predominant, (acinar 50%, papillary 20%, micropapillary 20% and lepidic 10%) |
| 28 | E426 | E19del | Study | Korean | male | Yes | ia | 0.8 | Minimally invasive adenocarcinoma (RUL_anterior seg) |
| 29 | E428 | E19del | Study | Korean | male | No | ia | 2.1 | Invasive adenocarcinoma, acinar predominant (acinar 65%, papillary 20%, micropapillary 10%, lepidic 5% ) |
| 30 | E429 | L858R | Study | Korean | male | Yes | ia | 1.7 | Invasive adenocarcinoma, lepidic predominant (lepidic 60%, acinar 40%) |
| 31 | E431 | L858R | Study | Korean | female | No | ia | 1.2 | Invasive adenocarcinoma, lepidic predominant, (lepidic 60%, papillary 40%) |
| 32 | E432 | L858R | Study | Korean | female | No | ia | 0.6 | Invasive adenocarcinoma, lepidic predominant (lepidic 60%, acinar 40%) |
| 33 | E434 | L858R | Study | Korean | female | No | ia | 10 | Invasive adenocarcinoma, lepidic predominant (lepidic 55% and acinar 45%) |
| 34 | E435 | E19del | Study | Korean | female | No | ia | 1.8 | Invasive adenocarcinoma, lepidic predominant (lepidic 70% and acinar 30%) |
| 35 | E436 | L858R | Study | Korean | female | No | ia | 1.96 | Invasive adenocarcinoma, lepidic predominant (lepidic 55% and acinar 45%) |
| 36 | E437 | L858R | Study | Korean | female | No | ia | 0.9 | Minimally invasive adenocarcinoma (lepidic 90%, acinar 10%) |
| 37 | E440 | E19del | Study | Korean | male | Yes | ia | 2 | Invasive adenocarcinoma, acinar predominant (acinar 80%, papillary 10%, lepidic 5%, micropapillary 5%) |
| 38 | E442 | L858R | Study | Korean | female | No | ia | 2.8 | Invasive adenocarcinoma, acinar predominant (acinar 55%, papillary 30%, micropapillary 10%, lepidic 5%) |
| 39 | E447 | E19del | Study | Korean | male | Yes | iib | 3 | Invasive adenocarcinoma, acinar predominant (acinar 65%, micropapillary 20%, papillary 10%, and lepidic 5%) |
| 40 | E449 | L858R | Study | Korean | female | No | ia | 1.4 | Invasive adenocarcinoma, lepidic predominant (lepidic 70%, acinar 25%, and papillary 5%) |
| 41 | E450 | E19del | Study | Korean | female | No | ia | 2.5 | Invasive adenocarcinoma, papillary predominant (papillary 50%, lepidic 30%, acinar 20%) |
| 42 | E452 | E19del | Study | Korean | female | No | ib | 3.1 | Invasive adenocarcinoma (acinar 55%, papillary 30%, micropapillary 10% and lepidic 5%) |
| 43 | E453 | L858R | Study | Korean | female | No | ia | 2.2 | Invasive adenocarcinoma, lepidic predominent (lepidic 60%, acinar 35% and micropapillary 5%) |
| 44 | E458 | L858R | Study | Korean | female | No | iia | 2.4 | invasive, acinar predominant, acinar 85%; lepidic 5%, solid 5%, and micropapillary 5%. Presence of signet ring cell features; 10% |
| 45 | E463 | E19del | Study | Korean | female | No | iia | 3 | invasive adenoca, mixed type, acinar and broncnioloalveolar type |
| 46 | E465 | E19del | Study | Korean | male | No | iiia | 3.5 | invasive papillary predominant, papillary 50%, acinar (cribriform) 30% and micropapillary 20%. |
| 47 | E470 | E19del | Study | Korean | female | No | iia | 3.7 | mixed type (acinar and papillary types) |
| 48 | E472 | E19del | Study | Korean | female | No | iia | 2.9 | mixed type (bronchioloalveolar and acinoar), moderately differentiated |
| 49 | E473 | E19del | Study | Korean | female | No | iiia | 3.7 | invasive mucinous adenocarcinoma, papillary 50%, acinar 30%, solid 10% an dlepidic 10% |
| 50 | E479 | E19del | Study | Korean | female | No | IIIA | 1.5 | predominantly acinar type |
| 51 | E482 | E19del | Study | Korean | female | No | iia | 2.2 | moderately differentiated, mixed subtype (acinar and bronchioloalveolar type) |
| 52 | E493 | L858R | Study | Korean | male | No | iia | 3.0 | acinar predominant (acinar, 60%; solid 20%, and micropapillary 10%) |
| 53 | E497 | L858R | Study | Korean | male | Yes | iiia | 2.5 | invasive, solid predominant, solid 70%, papillary 20%, and acinar 10% |
| 54 | E499 | E19del | Study | Korean | male | No | IIB | 3.1 | invasive, acinar predominant, acinar/cribriform 85% and micropapillary 15% |
| 55 | E502 | E19del | Study | Korean | female | No | iiia | 3.3 | mixed type(acinar, papillary and focal bronchioloalveolar) |
| 56 | E503 | E19del | Study | Korean | female | No | iiia | 2.7 | mixed type (solid and acinar variant) |
| 57 | E516 | L858R | Study | Korean | female | No | iiia | 3.5 | invasive, acinar predominant, acinar 40%, lepidic 30%, papillary 20%, and micropapillary 10% |
| 58 | E518 | L858R | Study | Korean | male | Yes | iia | 2.5 | invasive, micropapillary predominant pattern (micropapillary 60%, acinar 30%, lepidic 10%) |
| 59 | E522 | E19del | Study | Korean | female | No | iiia | 3 | invasive, micropapillary predominant(micropapillary 90%, and acinar 10%) |
| 60 | E523 | L858R | Study | Korean | male | Yes | iv | 2.5 | invasive, acinar predominant, acinar 60%, papillary 30%, micropapillary 10%. |
| 61 | E527 | L858R | Study | Korean | male | Yes | iiia | 2.8 | invasive, acinar predominant, acinar 40%, papillary 20%, lepidic 30%, and solid 10%. |
| 62 | E529 | E19del | Study | Korean | female | No | iia | 2.7 | invasive adenocarcinoma, acinar type, moderately differentiated |
| 63 | E532 | E19del | Study | Korean | female | No | iv | 2.2 | adenocarcinoma, invasive, acinar predominant, acinar 75%, papillary 10%, micropapillary 10% and solid 5% |
| 64 | E535 | E19del | Study | Korean | female | No | iia | 4.3 | mixed type, acinar and micropapillary |
| 65 | E538 | L858R | Study | Korean | female | No | iiia | 2.5 | invasive, micropapillary predominant, micropapillary 50%, acinar 30%, aolid 10% and lepidic 10% |
| 66 | E539 | L858R | Study | Korean | female | No | iiia | 3.5 | moderatly differentiated, mixed type (acinar, solid and papillary type) with lymphovascular invasion |
| 67 | E543 | L858R | Study | Korean | female | No | iiia | N.A | invasive, acinar predominant, acinar 50%, lepidic 20%, micropapillary 20%, and papillary 10% |
| 68 | E546 | L858R | Study | Korean | female | No | iiia | 2.1 | invasive, acinar predominant (70% acinar, 20% papillary and 10% micropapillary) |
| 69 | E547 | E19del | Study | Korean | female | No | iiia | 3.5 | mixed type (acinar, papillary and focal bronchioloalveolar) |
| 70 | E561 | E19del | Study | Korean | female | No | iiia | 2.6 | acinar type |
| 71 | E602 | L858R | Study | Korean | female | No | iib | N.A | acinar predominant |
